# Supplementary figures and images for: Fathers’ and Mothers’ support needs and support experiences after rapid genome sequencing
Source: Eur J Hum Genet. 2025 Dec 1;34(2):260–9. doi: 10.1038/s41431-025-01987-7 (PMC12858987; doi:10.1038/s41431-025-01987-7)

## Slide 1
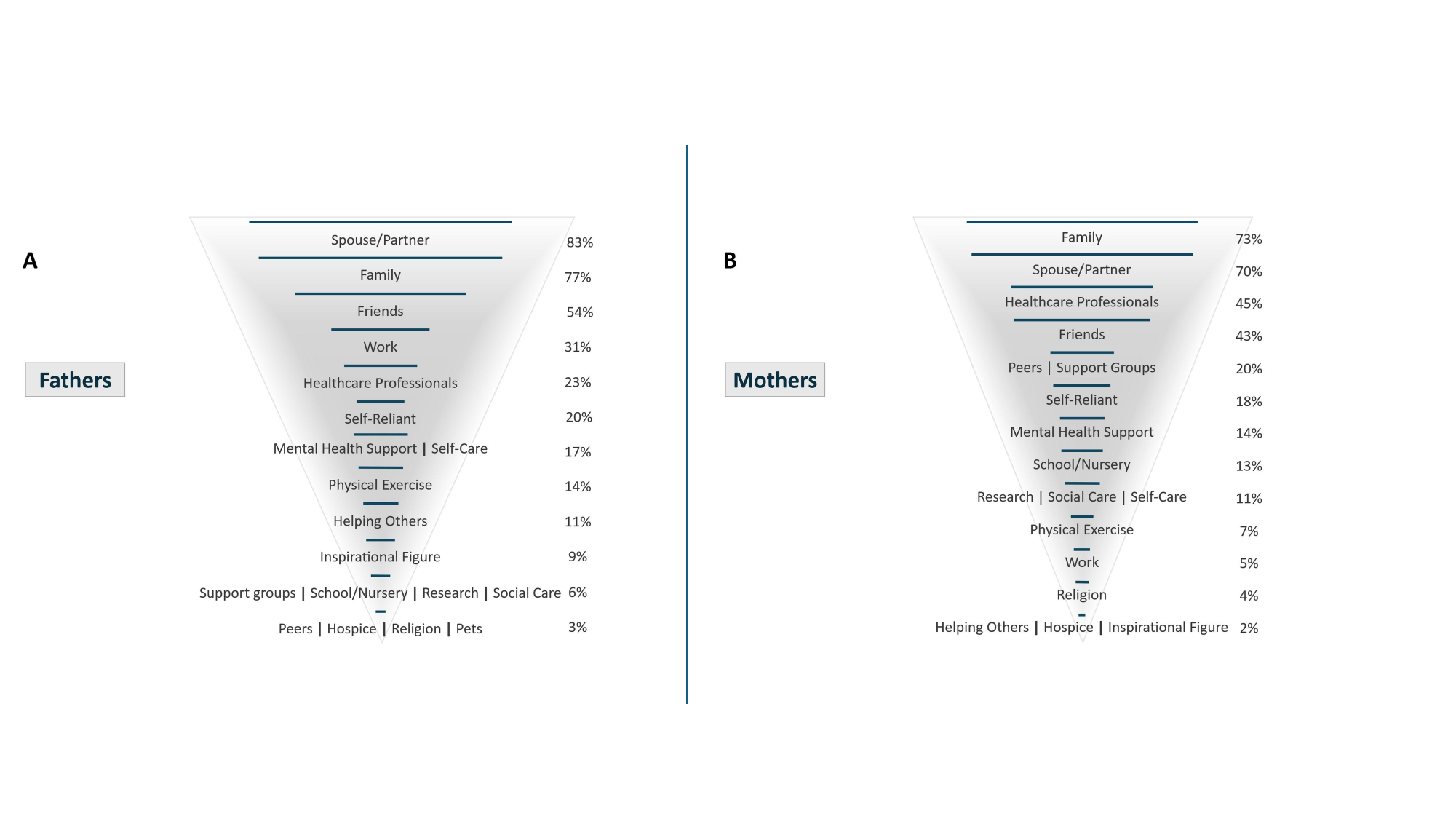

Supplement: Supplementary file 2 — Supplementary Figure 1 [file 41431_2025_1987_MOESM2_ESM.pptx]
